# Clinical Implications of Post-operative Haemoglobin Levels in Total Hip and Knee Arthroplasty

**DOI:** 10.7759/cureus.93533

**Published:** 2025-09-30

**Authors:** Phillip A McCarthy, Mohammad Mirza, Mohammed Arafa, Irrum Afzal, Georgina Thompson, Leila Mohammadi, Natalia Chance, Syed S Ahmed

**Affiliations:** 1 Trauma and Orthopaedics, Maidstone and Tunbridge Wells National Health Service (NHS) Trust, Royal Tunbridge Wells, GBR; 2 Orthopaedics, William Harvey Hospital, Ashford, GBR; 3 Orthopaedics, South West London Elective Orthopaedic Centre, London, GBR

**Keywords:** arthroplasty, day-case, haemoglobin, hip, knee, transfusion

## Abstract

Background: Current trends towards minimally invasive surgery and enhanced recovery encourage patients to mobilise and be discharged on the same day following total hip and knee arthroplasty (THA/TKA). The aim was to evaluate the value of current practice in measuring day 1 postoperative haemoglobin levels and stratify patient groups for whom this may be useful.

Methods: Three hundred and five patients who underwent elective THA or TKA, over six months (June-December 2023 inclusive), at a single trust, were included. Variables including age, sex, pre-operative and post-operative haemoglobin, American Society of Anaesthesiologists classification, approach, tourniquet time and the use of a drain were recorded. Odds ratio analysis was used to assess factors that may increase the risk of post-operative anaemia.

Results: A total of 157 THAs and 148 TKAs were included; 156/305 (51%) were found to have haemoglobin levels that would be defined as anaemic post-operatively. Risk factors identified corresponding to post-operative anaemia include female gender and pre-operative anaemia. The use of a drain in TKA was associated with a statistically significant increase in the drop in haemoglobin (-14.7 vs. -11.7 g/L, p = 0.034), whereas the use of a tourniquet and patient age made no statistically significant difference. In the THA cohort, the anterolateral approach resulted in a greater drop in haemoglobin than the posterior approach (-15.7 vs. -11 g/L; p = 0.017). Only one patient in each group underwent transfusion (0.6% THA, 0.7% TKA).

Conclusion: Our data show that drops in haemoglobin that require intervention are rare and therefore carrying out day 1 post-operative haemoglobin levels is unnecessary for most patients. Pre-operative patient variables such as pre-existing anaemia and female gender are predictive of risk.

## Introduction

In the UK, in 2023, 104,795 primary total hip arthroplasty (THA) and 88,163 primary total knee arthroplasty (TKA) procedures were performed [1]. Both total hip and knee replacements are mostly carried out in the management of end-stage osteoarthritis [2] but are also indicated in inflammatory arthropathies, avascular necrosis and trauma, with the end effect of alleviating pain and improving mobility. TKAs and THAs are both commonly performed and effective [3,4], with hip replacements among the most successful surgical procedures practiced in modern medicine, with 97% receiving a hip replacement under the care of the National Health Service reporting improvement in hip symptoms [5].

A growing and ageing population suggests that the demand for such procedures will increase substantially over the following decades, with the number of TKAs performed with the US population postulated to increase by 143% by 2050 compared to 2012 numbers [6]. In the UK, the numbers of THAs and TKAs are expected to increase by 37.7% and 36.6%, respectively [7]. The outpatient and inpatient care for these procedures is estimated to cost £9295 and £9482 for THAs and TKAs, respectively [8]. The average length of stay for both TKA and THAs is reported as 4.8 days. The unit cost at the point of delivery for an elective inpatient episode is £3749, with an additional £306 per additional bed day [9]. This contrasts with the unit cost of a day case procedure of £733 [9]. The inpatient stay, therefore, forms a substantial proportion of the financial burden of hip and knee replacement across the country [9].

The implementation of the enhanced recovery after surgery pathway has improved peri-operative patient care and has reduced lengths of stay. However, a review by Judge et al. did not demonstrate that this translated to improved cost-effectiveness [8]. The provision of day-case TKA, THA and unicompartmental knee replacement offers a promising option in reducing the financial burden in appropriately selected patients. It has been shown to be feasible in 15% of unselected cohorts without a corresponding increase in complications [10]. The overall length of stay is reduced [11], with a lower readmission rate following discharge [12]. Day-case hip and knee arthroplasty, defined as patient discharge on the day of surgery, is well-documented and provisioned internationally [13,14]. The uptake in the UK, however, remains low with 0.55% for THAs and 0.52% for TKAs. The national drive to safely increase numbers is accompanied by guidance from Getting It Right First Time, the British Association of Day Surgery and the Centre for Perioperative Care.

Among the parameters that limit the delivery of day-case arthroplasty surgery is the routine requirement to carry out post-operative blood tests on the first day following surgery. Although not a National Institute for Health and Care Excellence (NICE) recommended requirement following THA or TKA, this has become standard practice and is left to the clinicians’ discretion. Among the workup is the post-operative haemoglobin (Hb) level. This is generally used to screen for dangerously low post-operative Hb levels that may require transfusion. This is an important investigation as allogenic blood transfusion is associated with increased rates of surgical site infection [15,16], increased mortality following TKA [17] and increased length of stay following joint arthroplasty [18]. However, rates of post-operative transfusions have been reported at varying levels [19-22] with suggestions that improvements in operative techniques, adjuvant therapies and post-operative care are resulting in declining levels of blood loss and, therefore, declining transfusions [23,24]. An intervention that has had a significant impact on reducing intraoperative blood loss is the administration of tranexamic acid (TXA) peri-operatively [25,26], which is now part of the latest NICE guidance on primary hip and knee arthroplasty and should therefore be a standard procedure within the UK [27].

Recent trends in this area question the clinical necessity of these blood tests in all patients undergoing elective TKA and THA [28-32]. The focus of research into post-operative management is now moving towards developing a process of risk-stratifying patients at high risk of a greater post-operative drop in Hb, and therefore need for transfusion, in whom these blood tests would be clinically indicated [33-35]. Scoring systems such as the Total Hip Arthroplasty Blood Test Usefulness Score (THABUS) aim to use several variables to stratify patients.

An international consensus on post-operative anaemia has also identified that the true extent of a patient's post-operative anaemia does not become fully apparent until day 3 post-operatively [36], rendering the day 1 test a poor indicator of the true post-operative Hb.

The primary objectives of this study were to evaluate transfusion rates and patterns of Hb drop following THA and TKA, and to identify specific pre-operative and intraoperative risk factors predictive of significant post-operative anaemia needing intervention. This information aims to facilitate targeted patient management and support the development of a risk stratification tool. The primary outcome measure of this study was, therefore, the incidence of transfusion triggered by routine post-operative Hb checks. The secondary outcome measure was the drop in Hb seen on the first post-operative Hb results compared to the last pre-operative result.

Given the retrospective nature of the study, no formal power calculation was conducted, but the sample size was based on the available patient population within the defined timeframe.

This article was previously presented as an oral presentation at the 2024 Fred Heatley meeting on November 22, 2024, and the 2025 Sam Simmonds meeting on May 9, 2025.

## Materials and methods

A retrospective sample was collected comprising all patients who underwent elective THA or TKA at a single UK district general hospital from June to December 2023 inclusive. Patients were identified using national joint registry data, and further information, including blood results and the prescription of packed red cells, was taken from the trust's electronic patient record system. Each patient underwent pre-operative assessment, including a full blood count and measurement of Hb levels. Post-operatively, all patients had their Hb level taken the day following their procedure. Those patients not discharged on the first day post-operatively subsequently had their Hb levels checked daily.

Variables from the previously referenced literature, as well as those included in the THABUS, were recorded. These were patient age, sex, pre-operative Hb results, American Association of Anaesthesiologists (ASA) physical classification score, surgical approach, pre-operative anticoagulation, tourniquet time and the use of a drain. The surgical approaches used in THA were the posterior and anterolateral (Watson-Jones) approaches with the patient in a lateral decubitus position. For TKA, it was found that in our sample, only the medial parapatellar approach was used.

Despite the withholding of anticoagulation medication before arthroplasty in accordance with local hospital policy [37], data were collected for this variable within our sample. All patients were commenced on thromboprophylaxis following their surgery in line with local policy [38]. Drains were used selectively within the trust in patients undergoing TKA, but depending on intraoperative findings, some received a drain. The use of a tourniquet was, of course, only collected for patients undergoing TKA, and this was further subdivided into the length of time that the tourniquet was inflated. TXA was used as per NICE guidance, so it was not used as an inter-patient variable. TXA was administered to all patients peri-operatively according to current NICE guidelines.

The primary outcome was transfusion of packed red blood cells post-operatively during the same admission. Local transfusion guidelines were followed post-operatively. These advised threshold measures before red cell transfusion could be requested. These were an Hb of <70 g/L in patients with symptoms of acute anaemia and an Hb of <80 g/L in patients with a previous cardiac history. Further outcomes included post-operative anaemia (values defined by patient sex) and the total decrease in post-operative Hb compared to pre-operative levels. The diagnosis of anaemia was classified as an Hb of <130 g/L in men and <120 g/L in women.

An analysis of possible risk factors for a greater post-operative drop in Hb was carried out using a Mann-Whitney U test to assess the significance of the difference in Hb changes across groups. Odds ratio calculations were completed when assessing the risk of post-operative anaemia among groups. Analysis was performed using the MedCalc statistical software package (MedCalc Software Ltd., Belgium) [39].

There was no additional patient contact, and as such, this project was performed as a service evaluation and so was not submitted for formal ethical approval. The project was registered with and approved by the institution’s audit department and was conducted in accordance with the Declaration of Helsinki and the guidelines for Good Clinical Practice.

## Results

In total, 305 patients underwent elective THA or TKA during the period defined previously. Of these, 157 underwent THA and 148 underwent TKA. All patients had a preoperative Hb result as well as a post-operative sample taken on the first day after their operation. Within the total group, 156/305 (51%) were found to have Hb levels that would be defined as anaemic postoperatively (<130 g/L in men and <120 g/L in women). When divided by the operative procedure, this included 87 (55%) of THA patients and 69 (47%) of TKA patients.

With regard to the primary outcome of the study, within the entire THA cohort (n = 157), only one patient (0.6%) received packed red blood cells following surgery due to a symptomatic fall in their Hb to 76 g/L three days post-op. This patient’s pre-operative Hb was 132 g/L with a day 1 post-operative Hb of 100 g/L. Within the whole TKA cohort (n = 148), a total of one patient (0.7%) received packed red blood cells following surgery. This was due to a symptomatic drop of their Hb to 64 g/L three days post-op. This patient’s pre-operative Hb was 110 g/L with a day one post-operative Hb of 94 g/L.

For the entire THA cohort, the mean preoperative Hb was 135.7 g/L (range 103-166 g/L), and the mean postoperative Hb was 124 g/L (range 98 -157 g/L). The mean decrease in Hb levels following surgery was 11.6 g/L, representing a 9.4% decrease.

When comparing secondary outcome variables within the THA group, when comparing female patients (n = 94) and male patients (n = 63), the results showed that female patients undergoing THA experience a significantly larger decrease in Hb compared to male patients (-13 vs. -9 g/L, p = 0.0048). Additionally, a posterior approach gave a significantly smaller decrease in Hb compared to an anterolateral approach (-11.0 vs. 15.7 g/L, p = 0.035). Age, anticoagulation pre-operative and ASA grade showed no relation to the fall in Hb post-operatively. These comparisons are shown in Table 1.

**Table 1:** Mann-Whitney U analysis of the significance of difference in Hb drop within the THA cohort THA: total hip arthroplasty; ASA: American Association of Anaesthesiologists; Hb: haemoglobin

THA	Group 1	Group 2	U value	Z score	p value
Sex	Males (n = 63)	Female (n = 94)	2,174	-2.8170	0.0048
Approach	Antlat (n = 18)	Post (n = 136)	849	2.1060	0.0349
Anticoagulation	Yes (n = 33)	No (n = 123)	1,911	0.5121	0.6101
Age	≤70 (n = 92)	>70 (n = 65)	2,736.5	0.9016	0.3681
ASA	ASA 1 (n = 11)	ASA 3 (n = 24)	103	1.0127	0.3125

Risk factors that significantly increased the odds of developing post-operative anaemia in patients undergoing THA were an anterolateral approach (odds ratio, 3.30; 95% CI, 1.03-10.53; p = 0.04) and pre-operative anaemia (odds ratio, 25.55; 95% CI, 1.49-437.95; p = 0.03). Patient sex, age and pre-operative ASA score made no significant impact on developing post-operative anaemia. These comparisons are shown in Table 2.

**Table 2:** Odds ratio analysis of development of postoperative anaemia within the THA cohort THA: total hip arthroplasty; CI: confidence interval; ASA: American Association of Anaesthesiologists

THA	Group 1	Group 2	Odds ratio	95% CI	p value
Approach	Antlat (n = 18)	Post (n = 136)	3.30	1.0335-10.5372	0.0438
Pre-operative anaemia	Yes (n = 13)	No (n = 144)	25.55	1.4906-437.946	0.0254
Sex	Male (n = 63)	Female (n = 94)	0.73	0.3851-1.3911	0.3409
Age	<60 (n = 39)	>61 (n = 118)	1.20	0.5694-2.5496	0.6260
	<70 (n = 92)	>71 (n = 65)	1.85	0.8620-4.0013	0.1139
ASA	2 (n = 115)	3 (n = 24)	1.37	0.5569-3.3983	0.4894
Anticoagulation	Yes (n = 33)	No (n = 123)	1.67	0.7545-3.6816	0.2065

The mean preoperative Hb in the whole TKA cohort was 137.8 g/L (range 92-175 g/L), and the mean postoperative Hb was 125.6 g/L (range 81-159 g/L). The mean decrease in Hb level following surgery was 12.1 g/,L representing a 9.7% decrease in total Hb.

When looking at the factors affecting the change in Hb level within the TKA cohort, there was a significant difference in the decrease in Hb measurements in patients who had a drain inserted postoperatively (n = 23) and those that did not (n = 124) (-15 vs. -12 g/L, p = 0.034). Intra-operative tourniquet time, patient age, sex and preoperative ASA score made no significant impact on the change in a patient's Hb. This is shown in Table 3.

**Table 3:** Mann-Whitney U analysis of the significance of the difference in Hb drop within the TKA cohort TKA: total knee arthroplasty; ASA: American Association of Anaesthesiologists; Hb: haemoglobin

TKA	Group 1	Group 2	U value	Z score	p value
Drain	Yes (n = 23)	No (n = 124)	1,093.5	1.8181	0.0343
Tourniquet time	0-15 minutes (n = 17)	>30 minutes (n = 109)	788	-0.9854	0.3222
Sex	Male (n = 62)	Female (n = 86)	2,295.5	-1.4380	0.1499
ASA	ASA 2 (n = 76)	ASA 3 (n = 55)	1,821.5	1.2498	0.2113
Age	≤70 (n = 60)	>70 (n = 88)	2,328	-1.2166	0.2225

The only variable measured, which resulted in a significantly higher risk of developing post-operative anaemia in patients undergoing TKA, was pre-operative anaemia (odds ratio, 34.565; 95% CI, 2.005-595.792; p = 0.015). Patient sex, age (<70 vs. >70), the use of a drain, the use of anticoagulant medication pre-operatively and ASA score did not significantly increase the risk of patients developing post-operative anaemia. These comparisons are demonstrated in Table 4.

**Table 4:** Odds ratio analysis of the development of postoperative anaemia within the TKA cohort TKA: total knee arthroplasty; CI: confidence interval; ASA: American Association of Anaesthesiologists

TKA	Group 1	Group 2	Odds ratio	95% CI	p value
Sex	Males (n = 62)	Females (n = 86)	0.549	0.282-1.067	0.077
Pre-operative anaemia	Yes (n = 12)	No (n = 136)	34.565	2.005-595.792	0.015
Age	<70 (n = 60)	>70 (n = 88)	1.894	0.973-3.687	0.060
Drain	Yes (n = 23)	No (n = 125)	1.094	0.449-2.665	0.844
Anticoagulation	Yes (n = 38)	No (n = 110)	0.992	0.477-2.063	0.982
ASA	2 (n = 76)	3 (n = 55)	0.784	0.378-1.625	0.513

The odds ratio of developing post-operative anaemia was calculated for several variables with both the THA and TKA groups combined. Within this data set, there was a significantly increased risk of post-operative anaemia associated with pre-operative anaemia (odds ratio, 52.186; 95% CI, 3.135-868.70; p = 0.006) and an age above 70 years (odds ratio, 1.668; 95% CI, 1.036-2.560; p = 0.035). This is shown alongside the other variables assessed in Table 5.

**Table 5:** Odds ratio analysis of the development of postoperative anaemia within both the THA and TKA cohorts CI: confidence interval; ASA: American Association of Anaesthesiologists; THA: total hip arthroplasty; TKA: total knee arthroplasty

All patients	Group 1	Group 2	Odds ratio	95% CI	p value
Pre-operative anaemia	Yes (n = 22)	No (n = 283)	52.186	3.135-868.70	0.006
Age	<70 (n = 152)	>70 (n = 153)	1.668	1.036-2.560	0.035
ASA	2 (n = 191)	3 (n = 79)	1.381	0.815-2.340	0.230
Sex	Males (n = 125)	Females (n = 180)	0.649	0.410-1.028	0.065
Anticoagulation	Yes (n = 72)	No (n = 233)	1.213	0.714-2.060	0.476

It is important to note that while statistical significance was achieved in several comparisons, clinicians should evaluate these findings in the context of their practical clinical impact. For instance, the statistically significant drop in Hb associated with drain use in TKA warrants consideration of clinical protocols regarding drain placement.

## Discussion

This study aimed to assess the utility of routine post-operative Hb testing in patients undergoing elective THA and TKA. Our findings support the growing body of literature suggesting that routine early post-operative Hb monitoring may be redundant in most patients due to the exceedingly low transfusion rate observed (<1%), despite over half of the cohort demonstrating anaemia by standard laboratory thresholds. This aligns with recent studies, which indicate declining transfusion requirements, a trend attributable to improved surgical techniques and the standardised use of TXA, which has been shown to significantly reduce peri-operative blood loss and is now embedded within national guidance for arthroplasty practice [25,32]. Unfortunately, due to this low rate of transfusion, risk factors for the primary outcome measure could not be compared. Therefore, the analysis focused on the secondary outcome measures.

Our data support the concept that routine post-operative Hb checks may be of limited value in the general arthroplasty population, suggesting that targeted testing would benefit select subgroups. Notably, pre-operative anaemia emerged as a strong predictor of post-operative anaemia in both THA and TKA cohorts, with odds ratios exceeding 25 and 34, respectively. This underlines the importance of pre-operative optimisation and risk stratification. Furthermore, our analysis revealed a significantly greater drop in Hb in THA patients who underwent an anterolateral approach compared to those who underwent a posterior approach. It is essential to note that the choice of approach is, of course, surgeon-dependent, and this may have confounded the results, as the posterior approach is traditionally considered more aggressive.

The use of surgical drains in TKA was also associated with a significantly greater Hb drop, which is in keeping with previous studies. Drains have been linked to increased blood loss and higher transfusion rates, leading to their more selective use in contemporary arthroplasty practice [35,40]. Our findings corroborate those of Xu et al., who demonstrated higher transfusion rates (15.07%) and prolonged hospital stays in patients who received closed suction drainage following total joint arthroplasty, compared to those managed without drains (6.72%) [41].

It is noteworthy that age over 70 was independently associated with a higher risk of post-operative anaemia in the combined cohort. While the clinical relevance of anaemia in this group may vary, the finding supports targeted monitoring in older patients, who may have less physiological reserve to tolerate peri-operative blood loss.

The results stated above should, of course, be interpreted with an understanding of this study's limitations. In this case, the retrospective design and modest sample size limit the generalisability of findings. Furthermore, while statistically significant differences in Hb drop were observed, the study was underpowered to assess rare events such as transfusion-related complications or to explore interaction effects between multiple risk factors. Confounding variables, such as the surgeon's choice of approach for THA, may have produced the lower blood loss seen with the posterior approach. Nevertheless, the consistency of our results with those in the wider literature suggests the findings are clinically meaningful.

## Conclusions

Substantial falls in Hb after hip or knee arthroplasty are common, with 51% of patients being classified as anaemic with regard to their Hb levels the day following their procedure, with an overall decrease in Hb of 12 g/L. However, our data show that rates of transfusion following both THA and TKA are very low indeed. Despite the low transfusion rates, meaning primary outcome analysis could not be completed, significant differences were found within the secondary outcome analysis.

Regarding secondary outcome measures, variables have been identified that increase the risk of total Hb drop and post-operative anaemia in patients. These higher risk variables include an anterolateral surgical approach and pre-operative anaemia in patients undergoing THA, as well as the use of a drain and pre-operative anaemia in patients undergoing TKA. Overall, patients above 70 years of age were at a higher risk of post-operative anaemia. These factors could be used to risk-stratify patients undergoing THA and TKA electively to identify those more likely to become anaemic post-operatively and so should undergo post-operative day 1 Hb checks. In doing so, this would focus time and resources on those who would most benefit from repeat blood tests and a longer inpatient hospital stay. Further prospective studies with larger sample sizes are recommended to validate these findings and refine the proposed risk stratification criteria.

In summary, this study supports an approach to post-operative monitoring that is driven by risk stratification rather than routine. Patients with pre-operative anaemia, those undergoing THA via an anterolateral approach, TKA patients with drain insertion and individuals aged over 70 years may represent higher risk groups who may benefit from early post-operative Hb testing. Wider adoption of selective testing protocols may reduce unnecessary investigations, improve resource utilisation and facilitate safe expansion of day-case arthroplasty pathways without compromising patient safety.
